# Public Health Response to Tuberculosis Outbreak among Persons Experiencing Homelessness, Minneapolis, Minnesota, USA, 2017–2018

**DOI:** 10.3201/eid2603.190643

**Published:** 2020-03

**Authors:** Kelzee K. Tibbetts, Randy A. Ottoson, Dean T. Tsukayama

**Affiliations:** Hennepin County Public Health Department, Minneapolis, Minnesota, USA (K.K. Tibbetts, R.A. Ottoson, D.T. Tsukayama);; Hennepin County Medical Center, Minneapolis (D.T. Tsukayama)

**Keywords:** tuberculosis, contact tracing, homeless persons, public health response, homeless shelters, Minneapolis, Minnesota, USA, outbreak, *Mycobacterium tuberculosis*, persons experiencing homelessness, tuberculosis and other mycobacteria, respiratory infections, concentric-circle approach, bacteria

## Abstract

Tuberculosis (TB) is a greater risk for populations experiencing homelessness. When a TB exposure occurs in a homeless shelter, evaluation of contacts is both urgent and challenging. In 2017, local public health workers initiated a response to a TB outbreak in homeless shelters in Minneapolis, Minnesota, USA. In this contact investigation, we incorporated multiple techniques to identify, evaluate, and manage patients, including the concentric-circle method to characterize amount of contact, identifying the most frequent sites of sporadic medical care, using electronic medical records, and engaging with medical providers treating this population. Of 298 contacts evaluated, 41 (14%) had latent TB infection and 2 had active TB disease. Our analysis indicated a significant relationship between duration of exposure and positive TB test result (p = 0.001). We encourage local public health departments to expand beyond traditional contact tracing techniques by leveraging partnerships and existing systems to reach contacts exposed in shelters.

In 2017, a total of 9,093 new cases of active tuberculosis (TB) were reported in the United States, and ≈4.5% of these occurred in persons experiencing homelessness (PEH) in the year preceding their diagnosis (*1*). The incidence of TB in PEH populations is >10 times that of the general population (i.e., 36–47 vs. 2.8 cases/100,000 population during 2006–2010) (*2*) because risk factors, such as HIV infection, mental illness, substance abuse, and barriers to accessing healthcare, put them at higher risk. In addition, PEH often use homeless shelters and congregate in environments where the risk for TB transmission is greatly increased (*3*).

A priority for TB control and prevention is the screening of persons exposed to transmissible TB (*4*). Locating and fully evaluating contacts, essential components of a contact investigation, is difficult but especially urgent for controlling TB among PEH populations. Homelessness at the time of diagnosis indicates the need for a prompt contact investigation; however, guidance or consensus on how to identify contacts in these situations is lacking. Interviews of PEH persons with active TB are not always reliable sources for contact information. An analysis of ≈3,000 PEH and non-PEH TB patients in New York, New York, USA, demonstrated that experiencing homelessness in the year before diagnosis predicted the likelihood of that person’s contacts not being identified during interviews (*5*). Shelter rosters can also be incomplete and unreliable, often changing over the course of a single night.

Guidelines for contact prioritization in congregate settings have been established but are impractical to apply to large, complex populations because priority is assigned primarily by likelihood of infection and progression to active disease, which is difficult to determine in these settings (*6*). Mass screenings for latent TB infection (LTBI) eliminate the need for contact prioritization but are resource intensive and decrease the public health value of positive test results. In the absence of readily available information about a person’s susceptibility, duration of exposure to an infectious person has been used to prioritize contacts. Although this benchmark for exposure is linked to risk for transmission (*7*,*8*), the measure is not well defined.

In 2017, a total of 70 cases of TB were diagnosed in Hennepin County, the largest county in Minnesota, USA (population of 1.3 million). This area has experienced an ongoing cluster of genotypically linked TB cases among long-term PEH. The cluster, confirmed by whole-genome sequencing, could have been circulating in Hennepin County as early as 1992 (*9*) but was not identified until the Centers for Disease Control and Prevention (CDC) began providing TB genotyping services to state health departments in 2004. During 2008–2016, a total of 18 cases of this genotype cluster were identified, resulting in multiple complicated contact investigations (Figure 1, panel A).

**Figure 1:**
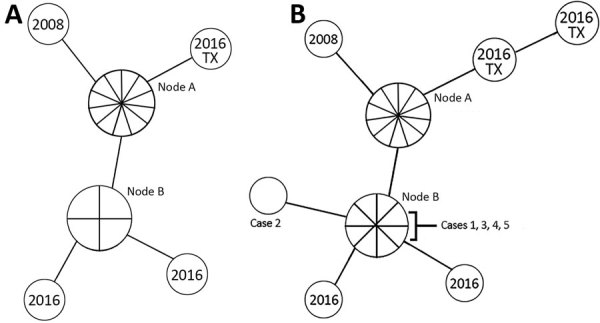
Whole-genome sequencing map of Hennepin County tuberculosis (TB) case cluster, Minneapolis, Minnesota, USA, including cases identified in Texas, USA, in 2016. A) 2008–2016 case cluster, which included 18 cases; B) updated 2008–2018 case cluster, totaling 24 cases. Isolates with the same genome sequence are displayed together in 1 node. Nodes are connected by lines proportional in length to the number of single-nucleotide polymorphism differences between isolates (n = 1, for all). No epidemiologic link to Minnesota was identified for the cases in Texas. Node A contains 10 cases diagnosed during 2008–2015 and the most recent common ancestor reference point. In panel A, node B contains 4 cases diagnosed during 2014–2016, and in panel B, node B contains 8 cases diagnosed during 2014–2018.

In 2016, Hennepin County Public Health (HCPH) was notified of 3 newly identified active TB cases matching the cluster previously found in its PEH population. The most infectious person had experienced coughing, night sweats, and weight loss, and testing revealed acid-fast bacilli (AFB)–positive sputum samples and a cavitary lesion on chest radiograph. The contact investigation for this person identified 180 shelter residents at high risk for infection in need of testing, 85% of whom were evaluated. 

In 2017, the HCPH was notified of another set of 3 newly identified active TB cases, which prompted another contact investigation. In this report, we review our experience conducting this contact investigation to prevent and control for a potential TB outbreak in Minneapolis homeless shelters during 2017–2018. We incorporated several methods that are not well described in the literature to identify, evaluate, and treat contacts. We specifically examined whether shelter rosters can be used to meaningfully quantify TB risk when multiple persons with TB have overlapping exposure periods and how outreach and partner strategies could be used to identify persons in need of TB testing.

## Characteristics of Case-Patients in 2017

In the spring of 2017, case-patient 1 was reported to HCPH and identified as having 48 nights of exposure to a person with active TB in a homeless shelter during the 2016 contact investigation. However, he had not been available for follow-up investigation. When located, he reported hemoptysis, had a cavitary lesion on chest radiograph, and was smear positive for TB (i.e., AFB-positive on sputum smears).

Case-patient 2 was also identified as a contact in the 2016 investigation and was fully evaluated >8 weeks after the date of last exposure; this case-patient had a negative interferon-γ release assay (IGRA) test result and a clear chest radiograph. Later in 2016, however, a repeat IGRA gave a positive test result, but LTBI treatment was deferred because of elevated liver enzymes and issues with compliance. Whether the IGRA conversion was caused by a subsequent exposure or an initial false-negative test result could not be determined. By the spring of 2017, this case-patient had findings on chest radiograph; no AFB was identified on sputum smears, but *Mycobacterium tuberculosis* was found on sample culturing.

Three weeks after the first 2 cases were diagnosed, a third was reported. Case-patient 3 had AFB-positive sputum smears, a noncavitary lesion on chest radiograph, and a cough complicated by untreated HIV. According to shelter rosters, case-patient 3 had brief shelter-based exposures to case-patients 1 and 2, but contact occurred around the time of case-patient 3′s symptom onset. No known exposure occurred between case-patient 3 and case-patients from the 2016 contact investigation. We identified only a weak epidemiologic link between case-patient 3 and the other case-patients identified in 2017, but whole-genome sequencing later confirmed that case-patient 3 was part of the same cluster.

## Methods

We interviewed case-patients about all sites of exposure during their infectious period, which was determined on the basis of their symptom onset and available clinical and radiologic evidence (*5*). Information gathered during these interviews suggested that exposures predominantly occurred at 3 homeless shelters in Minneapolis. Capacity of these shelters was 35–170 adult men per night; shelters had common sleeping areas, and no administrative controls were in place at these locations requiring TB screening at entry.

To determine the level of exposure within shelters, we informally collaborated with the Institute for Community Alliances, the Minnesota administrator for the Homeless Management Information System (HMIS), an electronic data collection tool that tracks services accessed by PEH. Staff of all single-adult shelters in Hennepin County manage client data with HMIS, providing complete information about shelter use. After receiving consent from case-patients, epidemiologists disclosed those patients’ names to HMIS staff, who compiled dates and locations of their shelter access and assembled full shelter rosters of all their contacts.

We adopted the concentric-circle approach for prioritizing contacts (*10*), using the number of nights of exposure to each case-patient (differing on the basis of perceived level of contagiousness) as a proxy for transmission risk; the first of the concentric circles included contacts with >10 days of exposure to smear-positive case-patients and >20 days exposure to smear-negative case-patients (Table 1). First-ring contacts underwent screening at a variety of locations (the Hennepin County Public Health Clinic and other county public health clinics, shelters, correctional facilities, hospitals, and primary care facilities) and were evaluated with either an IGRA or a tuberculin skin test (TST). If the initial TB test was conducted <8 weeks after the date of last exposure to the case-patient and the test results were negative, a second test was administered after >8 weeks had elapsed. All contacts, regardless of test results, were recommended to undergo chest radiography. Contacts who had symptoms or chest radiograph results compatible with active TB underwent clinical evaluation, and if indicated, they were asked to provide 3 sputum samples for smear, PCR, and culture testing. Persons with positive IGRA or TST results and for whom active disease was ruled out were encouraged to start LTBI treatment with daily isoniazid for 9 months, daily rifampin for 4 months, or once-weekly isoniazid/rifapentine for 12 weeks (*11*). Immunocompromised contacts and contacts previously treated for LTBI >10 years ago were also given physical examinations and the option to receive treatment for LTBI.

**Table 1:** Exposure criteria used to determine first concentric circle of contacts for case-patients 1–3 of Hennepin County tuberculosis case cluster, Minneapolis, Minnesota, USA, 2017

Case-patient no.	No. nights case-patient in shelter	Exposure criterion, no. nights
1	47	>10
2	28	>20
3	72	>10
1 and 3		>10

HCPH’s Healthcare for the Homeless program staff assisted with LTBI treatment case management by piloting a directly observed preventive therapy project in which they dispensed medication refills and tracked treatment at an on-site shelter clinic. To engage the medical community, HCPH relied on its collaboration with the Hennepin County Medical Center, a public hospital in Minneapolis. Because Hennepin County Medical Center is a safety-net healthcare facility, PEH regularly use its services and, therefore, have existing electronic health records (EHR). Of 890 adults identified by HMIS as accessing shelters for >2 weeks during September 2016–November 2017, a total of 84% had a Hennepin County Medical Center EHR. For first-ring contacts with a Hennepin County Medical Center EHR, we documented their exposure and the recommended screening procedures they needed to receive in their EHR and set a best-practice alert that would notify providers entering their record that the patient was a close TB contact. In addition, the alert also requested healthcare staff to update patient demographics and acquire patient contact information to provide HCPH epidemiologists. When the EHRs of these persons were accessed, epidemiologists received an immediate notification through the medical record system, providing real-time opportunities for communication between primary care and public health, if needed.

Last, we shared contact lists with relevant community partners, including shelter managers, staff at drop-in or advocacy centers, and clinic staff at jails and detoxification facilities. We asked partners to notify HCPH if contacts sought help at any of these locations.

After completing of the contact investigation, we analyzed outcome data by using the χ^2^ statistic to determine any relationship between duration of exposure and positive test results. We also reviewed how our methods contributed to contact identification and evaluation, captured transmission within a shelter setting, and facilitated LTBI treatment completion. Human subjects review for this synopsis was not required by our institutional review board, provided that the work involved a public health response, data were not traceable to individual patients, and informed consent was obtained from participants.

## Results

We identified 830 persons as having shelter-based exposure to the 3 TB case-patients identified in 2017; of these, 285 met the first-ring criteria for nights of contact and were recommended to undergo evaluation. Screening of these contacts began in late July 2017. By mid-September, 106 persons had completed evaluations; results for 78 were negative and 28 positive by either TB test administered. Of the contacts with positive test results, 10 had no recollection or documentation of prior testing, and 18 had histories of negative test results.

We identified 1 case of pulmonary disease (case-patient 4) during this early phase of screening. This US-born man had 32 nights of cumulative exposure to both smear-positive case-patients. He had a history of a negative TST result but during this investigation had a positive IGRA result and findings on chest radiograph. This case-patient reported a cough of <1 week and night sweats for 1–2 months. All 3 of his sputum samples were smear negative for AFB, but *M*. *tuberculosis* was confirmed by culture. Rapid genotyping data showed a minor difference with previous cases, but given whole-genome sequencing results and the strong epidemiologic link, he was included in the cluster, in consultation with CDC. Case-patient 4 accessed only 1 shelter during his infectious period.

An estimated 16%–31% of the PEH population in the United States has LTBI (*12*–*14*). During the 2016 shelter contact investigation, in which 180 contacts of smear-positive case-patients were identified, HCPH found a positivity rate of 23%. Given a preliminary positivity rate of 26% in our investigation, including 1 new case of disease, transmission exceeding the expected rate was evident; hence, we expanded our contact investigation into the second concentric circle of contacts.

First-ring contacts who were newly TB positive could all be epidemiologically linked to case-patient 1 during a short date range at a single shelter, the same (and only) shelter accessed by case-patient 4. Therefore, inclusion in the second ring was based on exposure to case-patient 1, and the exposure cutoff was decreased to >5 nights. Using these criteria, we identified 51 additional contacts.

Case-patient 5 had 5 nights of exposure to case-patient 1 and was evaluated in November 2017. He had a negative IGRA result, no TB symptoms, and findings on chest radiograph that had been stable since 2014. In August 2018, a follow-up radiograph showed more pronounced opacities, prompting a bronchoalveolar lavage, which was AFB smear negative but culture positive for *M*. *tuberculosis*. Genotyping of this case-patient’s isolate showed a match with the Hennepin County TB cluster (Figure 1, panel B).

In total, 338 first- and second-ring contacts were identified, and 298 (88%) were fully evaluated, a higher number than in other contact investigations done in this population (Table 2) (*6*,*15*). Of those evaluated, 227 had screening results that were negative for LTBI or active disease, 41 had positive test results but no disease (newly identified LTBIs), and 2 had positive TB test results and active disease and received TB diagnoses.

**Table 2:** Test results for first- and second-ring contacts, by number of nights of exposure to case-patient 1 of Hennepin County tuberculosis case cluster, Minneapolis, Minnesota, USA, 2017–2018*

No. nights exposed to case-patient 1	No. contacts	No. (%) contacts evaluated	Test results*		
			No. (%) positive	No. negative	No. other†
26–31	34	33 (97)	9 (27)	19	6
21–25	59	54 (92)	11 (20)	41	7
16–20	39	36 (92)	7 (19)	26	6
11–15	57	54 (95)	4 (7)	43	10
6–10	76	59 (78)	8 (14)	43‡	25
1–5	54	47 (87)	2 (4)	43	9
0	19	15 (79)	1 (7)	13	5
Total	338	298 (88)	42 (14)	228	68

*Contacts were tested by either the tuberculin skin test or interferon-γ release assay. †Contacts with history of a positive result by either tuberculosis test and those lost to follow-up. ‡Includes case-patient 5, who received an active tuberculosis diagnosis because his sputum was culture positive.

Percentage positivity trended downward as nights of exposure to case-patient 1 decreased. We analyzed data for a relationship between nights of exposure to case-patient 1 and TB test result by using the χ^2^ statistic and the cutoff of >15 nights of exposure and found a significant association (p = 0.001). The positivity rate of the high exposure group was 22%, and the positivity rate of the low exposure group was 9%. Overall, the positivity rate was 14%.

**Figure 2:**
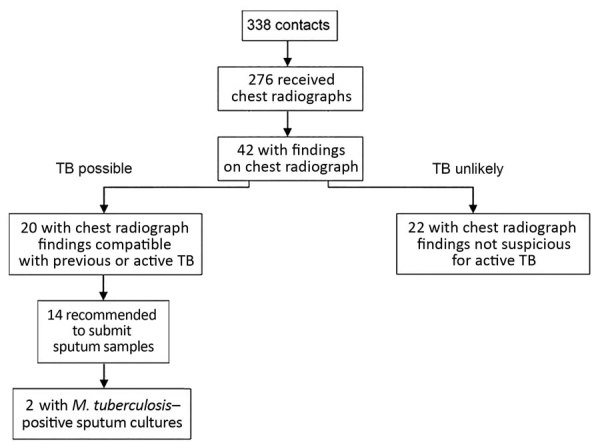
Chest radiograph findings and TB test results of contacts for case-patients 1–3 of Hennepin County TB case cluster, Minneapolis, Minnesota, USA, 2017–2018. At the initial screening, contacts were evaluated by either an interferon-γ release assay or a tuberculin skin test and recommended to undergo chest radiography. Those with suspected active TB were requested to provide sputum samples for further diagnostics (smear test, *Mycobacterium tuberculosis* culturing, and interferon-γ release assay). Two contacts were identified as having active pulmonary disease. TB, tuberculosis.

Chest radiographs were obtained for 276 contacts at the time of their TB screening (Figure 2). Healthcare providers noted findings on 20 radiographs and recommended sputum sample collection for 14 of these patients. Two (case-patients 4 and 5) were positive for *M*. *tuberculosis* by culture.

Treatment for newly identified LTBI was recommended to 41 contacts, and 32 started treatment. Of these, 21 (66%) completed treatment, 9 stopped treatment against medical advice, and 2 did not complete follow-up monitoring. The 3 LTBI patients who were treated through the Healthcare for the Homeless on-site directly observed preventive therapy clinic completed treatment.

## Discussion

This contact investigation was initiated after 3 genotypically related cases of TB were diagnosed among PEH and was expanded when 1 case of pulmonary disease and a high positivity rate were found. In total, 5 TB cases were identified. Our work demonstrates a relationship between duration of exposure and TB transmission and uses innovative techniques to identify, test, and treat contacts in homeless shelters.

In shelter environments, contact identification and prioritization are well-known challenges to a successful contact investigation (*13*). In our investigation, no close shelter contacts were identified during case-patient interviews, and bed rosters were not maintained in a way that was conducive to timely analysis. As an alternative to paper rosters kept by many shelter staff, the HMIS database provided electronic spreadsheets from which dates of stay, a proxy measurement for duration of contact, could be quantified. This database enabled HCPH staff to quickly identify persons at greatest risk for TB infection and begin locating them.

Using the concentric circle approach, we prioritized contacts on the basis of their duration of exposure to case-patients, setting first-ring inclusion thresholds at >10 nights for smear-positive case-patients and >20 nights for smear-negative case-patients. Most contacts in this contact investigation had exposure to multiple case-patients, and attributing infection to any 1 case-patient was not possible. However, the 2 secondary active TB cases and 40 of the 41 new LTBI cases were identified in contacts exposed to case-patient 1. The remaining LTBI patient (who had 16 nights of exposure to case-patient 3) was unable to recall ever having a past TB test but confirmed a history of homelessness and incarceration.

We found that degree of contagiousness was linked to transmission and that transmission generally decreased as nights of exposure to case-patient 1 decreased. This finding legitimizes our prioritization methods and demonstrates the value of focusing resources on the evaluation of contacts of highly contagious TB case-patients. The expansion of the contact investigation was also validated, as shown by the 8 cases of newly identified LTBI in persons with 6–10 nights of exposure to case-patient 1. Contacts in the second concentric circle might have had a high positivity rate because many were exposed to case-patient 1 in the days immediately preceding his diagnosis, when he was believed to be most contagious. Therefore, public health officials should consider not only the duration of exposure but also the timing of it.

Establishing relationships with healthcare services accessed by our contacts was essential for successful screening. EHRs conveyed exposure messages to providers and enabled epidemiologists to view historical TB testing and any treatment outcomes. The best practice alert, which contained instructions for evaluation, prompted screening for 33% of contacts. For enhanced contact tracing in future contact investigations, we recommend that public health departments learn where their contacts receive healthcare services and build strong partnerships with the staff at those locations.

PEH face many barriers when accessing healthcare, which were acknowledged as we developed screening strategies. IGRAs were the primary screening tool used in this investigation and are preferable to TSTs for groups that have historically low rates of returning to have their TSTs read (*16*). Likewise, we conducted chest radiography at the time of TB testing. According to CDC guidelines (*3*), chest radiographs would not have been obtained for contacts with negative TB tests and no symptoms unless the persons were known to have concurrent medical conditions that increased risk (e.g., HIV, diabetes mellitus, substance abuse). Chest radiographs provided additional information that contributed to the diagnosis of active disease in 1 contact (case-patient 5). In addition, with this population’s high risk for TB exposure, the baseline films obtained become of great value in future contact investigations. Additional measures to reduce healthcare barriers included the Hennepin County Public Health Clinic offering incentives to contacts for completing screening tests and, later, the Healthcare for the Homeless staff facilitating annual IGRAs for shelter residents.

LTBI treatment completion is one of the best interventions for preventing cases of TB disease, but homelessness has been found to be significantly associated with incomplete treatment (*17*). We used 2 solutions to help contacts complete their treatments. First, the Healthcare for the Homeless project demonstrated that intensive case management combining medication tracking, dispensing, and education can prevent treatment failure. When using directly observed preventive therapy for patients at high risk for noncompliance along with the 12-week regimen, we saw 80% treatment completion, a level similar to that seen in a large-scale analysis of treatment completion among PEH (*18*).

Public health investigations in homeless shelters are not without their limitations, the most critical of which is locating contacts for evaluation. We were unable to complete follow-up for nearly 12% of our contacts were lost to follow-up, some of whom were accessing shelters outside of Hennepin County. HMIS is used statewide, but flagging records with exposure information was ultimately deemed too time consuming and had privacy concerns. EHRs can be useful to TB investigators and should be used to their full potential while still considering patient confidentiality and the urgency of a contact investigation. In addition, although partnering with shelter and day center staff provided opportunities for TB education and demonstrated transparency, these partnerships ultimately yielded no contacts.

Epidemiologists received no verification from case-patients about their exposures to contacts in shelters, and we could not exclude exposures that contacts might have had to these case-patients outside the shelter setting. Likewise, the results here were reported under the assumption that case-patient 1 had the only case with transmission potential. However, exposure to additional unidentified persons with TB infections might have occurred. When prioritizing contacts, we also did not give weight to the shelter environments (e.g., ventilation, bed proximity, and congregate areas within the shelter) of the facilities affected by the outbreak, although these factors could have affected transmission (*19*).

Despite our comprehensive approach to treating new cases of LTBI, treatment completion was low overall, a challenge not unique to this study (*20*,*21*). Although our work can inform contact identification, prioritization, and screening methods, unless LTBI treatment completion rates improve, which requires intensive resources, we expect to see a continuation of this TB cluster in Hennepin County in the PEH population.
